# Bedside Bioimpedance and Simple Functional Tests Identify Muscle Function Impairment Predicting Overall Survival in Chemotherapy-treated Metastatic Urothelial Carcinoma: A Retrospective Pilot Study

**Published:** 2026-09-01

**Authors:** NOBUHITO MURAMOTO, KOSUKE KITAMURA, TOMOYA SHIRAKAWA, KAZUHIRO FUKUDA, AKIMASA KURE, TOSHIYUKI CHINA, HARUNA KAWANO, SHUJI ISOTANI, SATORU MUTO, HISAMITSU IDE, SHIGEO HORIE

**Affiliations:** 1 Department of Urology, Juntendo University, Graduate School of Medicine, Tokyo, Japan; 1 Department of Urology, Juntendo University, Graduate School of Medicine, Tokyo, Japan; 2 Human Disease Models, Institute of Laboratory Animals, Tokyo Women's Medical University, Tokyo, Japan; 2 Human Disease Models, Institute of Laboratory Animals, Tokyo Women's Medical University, Tokyo, Japan; 3 Department of Advanced Informatics for Genetic Diseases, Juntendo University, Graduate School of Medicine, Tokyo, Japan; 3 Department of Advanced Informatics for Genetic Diseases, Juntendo University, Graduate School of Medicine, Tokyo, Japan; 4 Department of Urology, Juntendo University Nerima Hospital, Tokyo, Japan; 4 Department of Urology, Juntendo University Nerima Hospital, Tokyo, Japan; 5 Department of Digital Therapeutics, Juntendo University, Graduate School of Medicine, Tokyo, Japan; 5 Department of Digital Therapeutics, Juntendo University, Graduate School of Medicine, Tokyo, Japan

**Keywords:** bioimpedance analysis, chemotherapy, dynapenia, metastatic urothelial carcinoma, sarcopenia

## Abstract

**Objectives:**

To evaluate whether bedside bioimpedance analysis and simple functional tests can identify muscle function impairment associated with overall survival in chemotherapy-treated metastatic urothelial carcinoma.

**Materials:**

Retrospective single-center observational cohort study.

**Methods:**

We reviewed 56 consecutive patients treated with systemic chemotherapy between January 2014 and February 2021. Skeletal muscle index was estimated using bioimpedance analysis. Handgrip strength and walking speed were assessed in routine care. Sarcopenia-related categories were defined using sex-specific Asian Working Group for Sarcopenia thresholds. The prespecified primary comparison was reference (normal plus pre-sarcopenia) versus muscle function impairment (dynapenia plus sarcopenia). Overall survival was evaluated using Kaplan-Meier analysis and Cox models with minimal age adjustment.

**Results:**

Muscle function impairment was associated with shorter overall survival compared with the reference group. The association remained after age adjustment. Baseline characteristics suggested greater vulnerability in the impairment group, including lower body weight and serum albumin and higher inflammatory burden.

**Conclusions:**

A pragmatic bedside phenotype based on bioimpedance analysis and simple functional testing may help identify chemotherapy-treated metastatic urothelial carcinoma patients at higher risk of poor survival.

## Introduction

Metastatic urothelial carcinoma (mUC) remains a lethal malignancy despite advances in systemic therapy. Treatment decisions in mUC are frequently influenced by patient fitness, comorbidity, and tolerance to chemotherapy. However, routine clinical assessments may not capture the multidimensional vulnerability often described as frailty.

Sarcopenia refers to a loss of skeletal muscle mass and function and has been linked to adverse outcomes in multiple cancers^1, 2)^. Importantly, contemporary consensus frameworks emphasize that muscle function, including strength and physical performance, is not merely a correlate of muscle mass but a distinct and clinically meaningful domain^3, 4)^.

Dynapenia, defined as reduced muscle strength with preserved muscle quantity, may represent an earlier or different pathway of functional decline^5, 6)^. In geriatric medicine, strength-based phenotypes predict disability and mortality more robustly than muscle mass alone^7, 8)^.

In mUC, several studies have associated CT- derived measures of skeletal muscle, such as psoas area or the L3 cross-sectional muscle index, with survival and treatment toxicity^9, 10)^. While CT is widely available, muscle segmentation requires dedicated time and software and is not uniformly incorporated into routine practice. Bioimpedance analysis (BIA) offers a noninvasive and rapid estimate of body composition and can be performed at the bedside or outpatient clinic^11, 12)^. Additionally, the reliability of InBody devices has been reported in comparison with reference modalities such as DXA^13)^. When combined with simple functional measures such as grip strength, BIA-based assessment could provide a feasible screening approach to identify vulnerable patients^3, 4)^.

We therefore conducted a retrospective pilot study to evaluate whether sarcopenia-related categories assessed by BIA and grip strength are associated with overall survival in mUC patients treated with chemotherapy. We hypothesized that categories reflecting impaired muscle function (dynapenia and sarcopenia) would demonstrate poorer survival compared with patients with preserved function.

## Materials and Methods

### Study design and setting

This retrospective observational study included consecutive patients with metastatic urothelial carcinoma treated with systemic chemotherapy at Juntendo University Hospital between January 2014 and February 2021. The study was approved by the Institutional Review Board of Juntendo University Hospital (approval number: 15-005) and conducted in accordance with institutional ethical standards for clinical research involving human participants. Written informed consent was obtained from all participants after providing an oral explanation of the study.

### Participants

Eligible patients had pathologically confirmed urothelial carcinoma with metastatic or unresectable disease and underwent baseline body composition assessment prior to or at initiation of systemic chemotherapy. Patients without the baseline BIA assessment or without survival follow-up data were excluded from survival analyses.

### Body composition assessment

Body composition was evaluated using a multi- frequency segmental bioimpedance analyzer (InBody 770)^11-13)^. Parameters extracted included body weight, body mass index (BMI), total muscle mass, and segmental muscle mass. Skeletal muscle index (SMI) and sarcopenia-related classification were determined from the device outputs using sex-specific thresholds aligned with the Asian Working Group for Sarcopenia (AWGS) consensus approach^3)^.

### Muscle function assessment

Handgrip strength was assessed as part of routine clinical evaluation and classified as low or normal using sex-specific cutoffs consistent with AWGS recommendations^3)^. Walking speed was also assessed for all patients at baseline as part of routine functional evaluation. We used grip strength, walking speed, and SMI to classify patients into the four sarcopenia-related categories as defined in Table S1.

### Definitions of sarcopenia-related categories

Dynapenia is defined as reduced muscle strength that is not explained by low muscle quantity or mass^5, 6)^. Sarcopenia-related categories were defined using sex-specific thresholds for grip strength, walking speed, and skeletal muscle index (SMI), as summarized in Table S1. Patients were categorized as normal, pre-sarcopenia, dynapenia, or sarcopenia, corresponding to preserved function with normal SMI, low SMI with preserved function, low function without low SMI, and low SMI with low function, respectively. For inferential analyses, the primary comparison was prespecified as reference (normal+pre-sarcopenia) vs muscle function impairment (dynapenia+sarcopenia) to avoid unstable inference from very small subgroups.

Keywords: metastatic urothelial carcinoma; chemotherapy; bioimpedance analysis; sarcopenia; dynapenia; handgrip strength; frailty; overall survival

### Outcome and follow-up

Baseline assessment was defined as the date of the body composition and functional evaluation performed immediately before or at initiation of first-line systemic chemotherapy. Overall survival (OS) was calculated from the date of baseline assessment to death from any cause. Patients who were alive at last follow-up were censored on that date.

### Statistical analysis

Continuous variables are summarized as median (interquartile range [IQR]) and compared with the Mann-Whitney U test. Categorical variables are summarized as counts (percentages) and compared with Fisher’s exact test. OS was estimated using the Kaplan-Meier method and compared with log- rank testing. Cox proportional hazards regression was used for univariate analysis and for an age- adjusted model as a minimal prespecified adjustment. Given the limited number of events and the small sizes of dynapenia and sarcopenia groups, extensive multivariable modeling was avoided to reduce overfitting; exploratory models separating dynapenia and sarcopenia are presented as sensitivity analyses. Statistical analyses were performed using GraphPad Prism 10 (GraphPad Software, San Diego, CA, USA) and R, and cross-checked in Python. This study was designed as a hypothesis- generating pilot study; therefore, no a priori sample size calculation was performed and the statistical power was limited.

## Results

### Study cohort and classification

A total of 56 patients with metastatic urothelial carcinoma who underwent baseline body composition assessment were included in the survival analysis. Patients were categorized into four sarcopenia- related groups based on skeletal muscle index and handgrip strength: normal (n = 30), pre-sarcopenia (n = 19), dynapenia (n = 4), and sarcopenia (n = 3) (Figure 1). For inferential analyses, we prespecified a binary grouping to improve statistical stability: reference (normal + pre-sarcopenia) versus muscle function impairment (dynapenia + sarcopenia).

**Figure 1:**
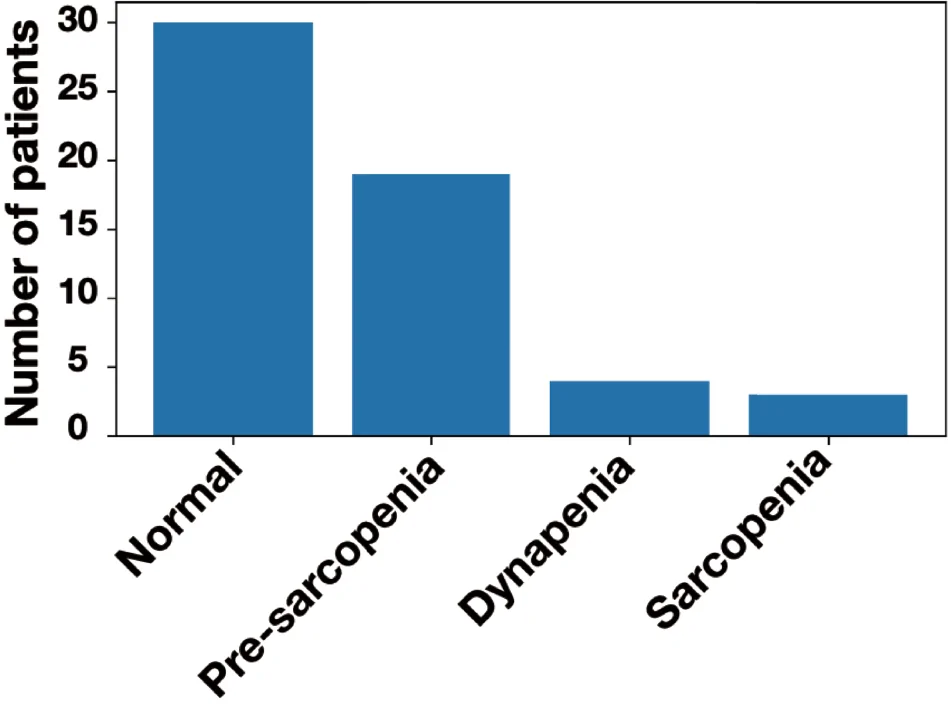
Distribution of sarcopenia-related categories Patients were classified as normal, pre-sarcopenia, dynapenia, or sarcopenia.

### Baseline characteristics

Baseline characteristics stratified by the prespecified binary grouping are summarized in Table 1. Overall, patients in the muscle function impairment group tended to be older than those in the reference group. As expected from the classification criteria, grip strength and several measures of body composition differed across groups. In this cohort, the impairment group showed lower body weight and serum albumin and tended to have higher C-reactive protein, suggesting greater vulnerability. We also report laboratory indices related to systemic inflammation and nutritional status, including complete blood counts, serum albumin, and inflammatory markers when available, to provide clinical context for disease burden and host condition. Collectively, these findings suggest that impaired muscle function co-occurred with features consistent with frailty-related vulnerability in this cohort. In addition, baseline body composition and selected laboratory indices stratified by the four sarcopenia- related categories are shown in Figure S1 for descriptive visualization of between-group distributions.

**Table 1:** Baseline patient characteristics

	Total (N = 56)	Normal+Presarcopenia (N = 49)	Dynapenia+Sarcopenia (N = 7)	*p*-value
Sex, n (%)				0.424
Male	35 (63)	33	2	
Female	21 (37)	16	5	
T stage, n (%)				0.371
T ≦ 2	38 (70)	33	5	
T > 2	18 (30)	16	2	
Primary site, n (%)				0.037
Bladder	30 (54)	28	2	
Upper urinary tract	26 (46)	21	5	
Resection of primary site, n (%)				0.072
Yes	37 (66)	34	3	
No	19 (34)	15	4	
First-line chemotherapy, n (%)				0.174
Gemcitabine plus Cisplatin	47 (84)	43	4	
Gemcitabine plus Carboplatin	6 (11)	4	3	
Other	3 (5)	2	0	
Charlson Comorbidity Index (CCI), n (%)				
< 3	26 (46)			
≥ 3	30 (54)			
Age and functional measures				
Demographics and functional measures, median (IQR)				
Median age at diagnosis (years) (IQR)	70 (65-75)	69 (64-75)	73 (70-80)	0.219
Handgrip strength (kg) (IQR)	28.7 (23.7-34.0)	30.4 (24.7-34.8)	17.9 (17.0-23.7)	0.047
10m-walking speed (m/sec) (IQR)	1.18 (1.04-1.33)	1.21 (1.04-1.34)	1.06 (1.00-1.18)	0.563
Body composition (IQR)				
BMI (kg/m^2^)	22.1 (19.7-24.8)	22.5 (19.8-24.9)	20.3 (17.1-22.3)	0.109
Body weight (kg)	59.0 (49.1-68.4)	61.3 (50.8-69.2)	45.7 (43.6-49.8)	0.078
Lean body mass (kg)	42.6 (34.1-48.4)	43.7 (35.9-49.0)	35.6 (32.6-37.9)	0.938
Body fat mass (kg)	15.3 (9.5-18.2)	17.2 (12.4-20.5)	9.9 (5.9-13.7)	0.031
Body fat rate (%)	25.8 (16.5-28.6)	25.9 (20.9-31.3)	21.4 (14.3-27.7)	0.063
SMI (kg/m^2^)	6.8 (6.0-7.7)	6.9 (6.0-7.8)	6.3 (5.5-6.9)	0.578
Phase angle (°)	4.4 (4.1-4.9)	4.5 (4.2-4.8)	4.2 (3.4-5.3)	0.661
Laboratory data (IQR)				
Leukocytes (cells/μL)	5800 (4975-7100)	5800 (5000-7100)	5500 (4850-7850)	0.578
Neutrophils (cells/μL)	3926 (3596-4402)	3897 (3584-4326)	4280 (3752-4477)	0.156
Lymphocytes (cells/μL)	1302 (955-1604)	1380 (962-1618)	1009 (968-1165)	0.109
Hemoglobin (g/dL)	12.7 (11.4-13.9)	12.8 (11.6-14.0)	11.5 (10.1-13.3)	0.668
Albumin (g/dL)	4.0 (3.7-4.1)	4.0 (3.8-4.1)	3.5 (3.4-3.6)	0.016
eGFR (mL/min/1.73 m^2^)	65.1 (52.0-81.9)	65.4 (52.4-81.8)	53.6 (40.2-69.1)	0.469
C-reactive protein (mg/dL)	0.25 (0.10-0.40)	0.10 (0.01-0.16)	0.80 (0.15-1.65)	0.297

Baseline demographics and clinical characteristics of the overall cohort and stratified by sarcopenia-related grouping: reference (normal + pre-sarcopenia) versus muscle function impairment (dynapenia + sarcopenia). Values are shown as n (%) unless otherwise specified.

### Overall survival

During follow-up, overall survival differed between the prespecified binary groups. Kaplan-Meier analysis demonstrated significantly shorter OS in the muscle function impairment group compared with the reference group. Median OS was 80.6 months in the reference group versus 21.0 months in the impairment group (*p* = 0.0165) (Figure 2). The separation of survival curves was most pronounced early after baseline assessment, consistent with the observation that functional impairment may capture near-term vulnerability among patients receiving systemic chemotherapy.

**Figure 2:**
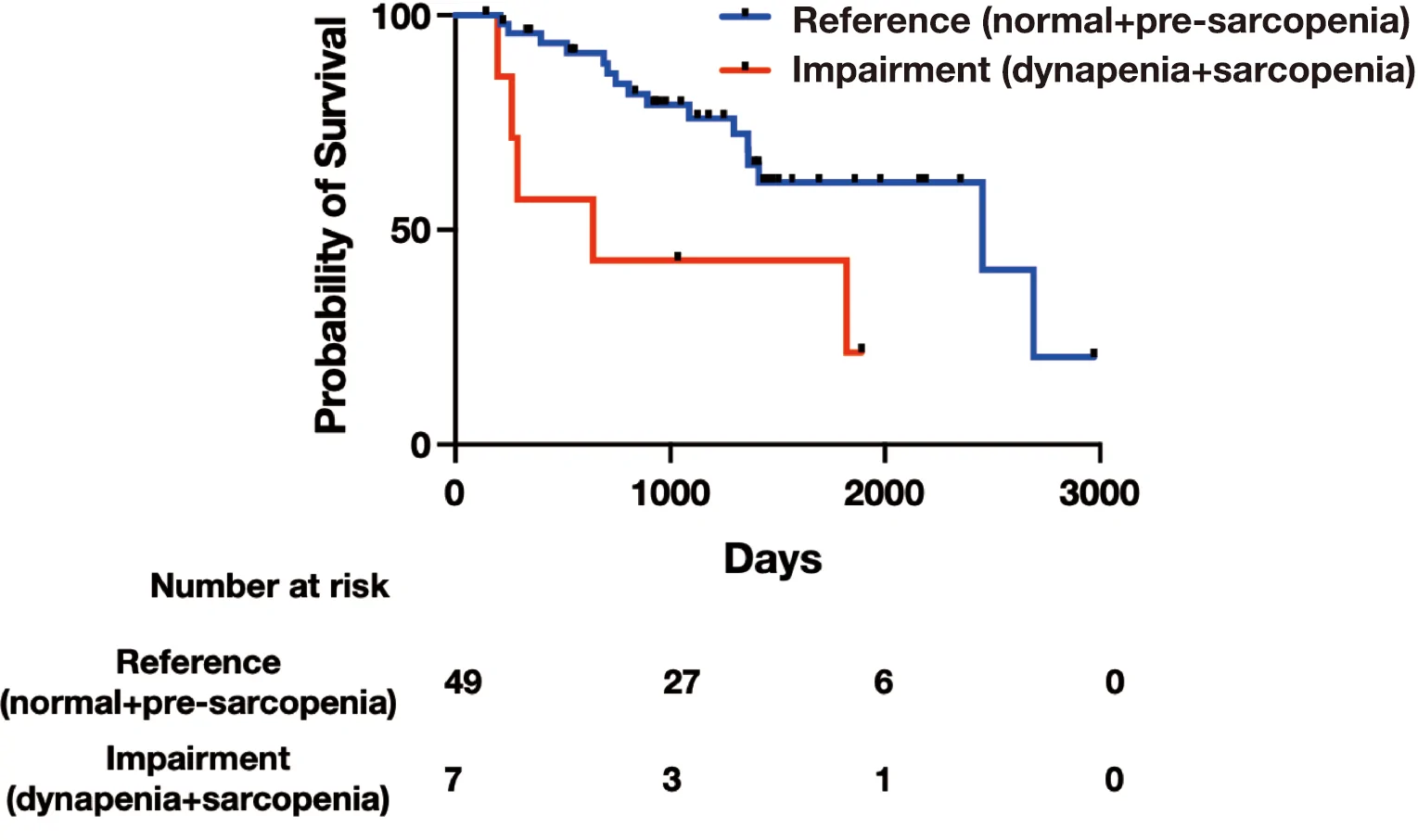
Kaplan-Meier overall survival by muscle function impairment Kaplan-Meier curves compare overall survival between the reference group (normal + pre-sarcopenia) and the muscle function impairment group (dynapenia + sarcopenia). Numbers at risk are shown below the x-axis.

### Cox regression and minimal adjustment

In Cox proportional hazards regression, muscle function impairment was associated with a higher hazard of death. In univariate analysis, impairment was associated with an approximately threefold increased risk of mortality (HR 3.29, 95% CI 1.17-9.20; p = 0.024). To reduce overfitting given the limited number of events, we prespecified a minimally adjusted model including age only. The association remained statistically significant after age adjustment (HR 2.92, 95% CI 1.01-8.47; p = 0.048), indicating that impaired muscle function retained prognostic information beyond age alone (Table 2).

As a sensitivity analysis, we further separated dynapenia and sarcopenia while retaining the same reference category (normal + pre-sarcopenia). Both dynapenia and sarcopenia demonstrated elevated hazard ratios relative to the reference group (dynapenia HR 2.82; sarcopenia HR 3.08). However, confidence intervals were wide, reflecting the very small subgroup sizes (n = 4 and n = 3, respectively), and these estimates should be interpreted as exploratory (Table 2).

**Table 2:** Cox proportional hazards models for overall survival

	HR	95% CI	*p* value
Univariate model			
Muscle function impairment (dynapenia+sarcopenia vs normal+pre-sarcopenia)	3.29	1.17-9.20	0.024
Age-adjusted model (age in years)			
Muscle function impairment (dynapenia+sarcopenia vs normal+pre-sarcopenia)	2.92	1.01-8.47	0.048
Dynapenia (vs normal+pre-sarcopenia)	2.82	0.78-10.23	0.115
Sarcopenia (vs normal+pre-sarcopenia)	3.08	0.68-13.95	0.143

Cox proportional hazards regression models evaluating the association between muscle function status and overall survival are shown. The primary comparison contrasts muscle function impairment (dynapenia + sarcopenia) with the reference group (normal + pre-sarcopenia). The age-adjusted model includes age (years) as the only covariate to minimize overfitting in this pilot cohort. Exploratory analyses separately estimate hazard ratios for dynapenia and sarcopenia versus the reference group; these subgroup estimates should be interpreted cautiously due to small sample sizes. Hazard ratios (HRs) are presented with 95% confidence intervals (CI).

### Exploratory four-group analysis

Kaplan-Meier curves across the four sarcopenia-related categories (normal, pre-sarcopenia, dynapenia, and sarcopenia) are shown in Figure S2 for descriptive purposes. Interpretation is limited by the small numbers of patients in the dynapenia and sarcopenia groups. Although the global comparison across four groups suggested differences in survival, pairwise comparisons with multiple-testing adjustment did not identify statistically significant differences between individual categories, likely due to limited statistical power and sparse events in the dynapenia and sarcopenia groups. Therefore, the four-group analysis is presented as exploratory and hypothesis-generating, supporting the prespecified binary grouping as the primary inferential framework in this pilot dataset.

## Discussion

This retrospective pilot study suggests that sarcopenia-related phenotypes characterized by impaired muscle function are associated with shorter OS among chemotherapy-treated patients with metastatic urothelial carcinoma. The association was evident using a pragmatic binary grouping that combined dynapenia and sarcopenia and remained significant after minimal adjustment for age. Given the small sample size and the exploratory nature of the analyses, these findings should be interpreted as hypothesis-generating; however, the direction and magnitude of effect were consistent across models.

Prior work in urothelial carcinoma has mainly evaluated CT-derived measures of skeletal muscle quantity, such as psoas area or L3 muscle index, and has linked low muscle mass to survival and treatment-related toxicity^9, 10)^. Our results complement this literature by focusing on the functional component of sarcopenia. Contemporary consensus frameworks emphasize that muscle strength and physical performance are clinically meaningful domains that do not always parallel muscle quantity^3, 4)^. In this context, grip strength may capture multidimensional vulnerability that reflects frailty and limited physiologic reserve, and may therefore provide prognostic information beyond body composition alone^3, 4)^. The concept of dynapenia further supports this distinction, highlighting reduced muscle strength as a clinically relevant phenotype that may occur even when muscle quantity is relatively preserved^5, 6)^.

From a clinical perspective, the approach tested here is feasible for routine practice. BIA can be obtained rapidly without radiologic segmentation, is noninvasive, and can be repeated over time, making it suitable for outpatient or bedside assessment^11, 12)^. InBody-based estimates have also been reported to show acceptable agreement with reference methods such as DXA in validation studies^13)^. If validated in larger cohorts, incorporating function-based screening into baseline evaluation could help identify patients who may benefit from proactive supportive care, such as nutrition counseling, resistance exercise programs, rehabilitation interventions, and closer monitoring during chemotherapy^3, 4)^.

Compared with CT-based sarcopenia assessment, which typically requires muscle segmentation with dedicated software and trained personnel time, the bedside approach used here can be completed in routine care without additional imaging or post-processing^11, 14)^. This pragmatic difference may facilitate broader implementation and repeated assessment if measurement procedures are standardized across institutions.

Several mechanisms may underlie the observed association between muscle function impairment and worse survival. Reduced strength can reflect systemic inflammation, malnutrition, cancer cachexia, and decreased physical activity, all of which can diminish physiologic reserve and impair the ability to tolerate cytotoxic therapy^8)^. Laboratory measures presented in Table 1 provide additional context for host condition and systemic disease burden, although the retrospective design does not allow causal inference and residual confounding is likely.

This study has strengths that support its clinical relevance. We evaluated a pragmatic assessment strategy using routinely obtainable measures in a real-world cohort and prespecified a binary comparison to improve interpretability and reduce instability from very small subgroups. We also presented exploratory analyses separating dynapenia and sarcopenia to improve transparency, while acknowledging that these estimates are imprecise due to limited sample size.

Several limitations should be noted. This was a retrospective single-center study with a small number of patients in the dynapenia and sarcopenia categories, limiting statistical power and restricting multivariable adjustment. Because the dynapenia and sarcopenia subgroups were very small, this work should be interpreted strictly as hypothesis- generating, and its statistical power to detect subgroup effects is limited. An additional potential confounder is the imbalance in primary tumor site between groups. Upper urinary tract urothelial carcinoma and bladder cancer can differ in clinical behavior and prognosis, and the higher proportion of upper urinary tract tumors in the impairment group may have contributed to the observed survival difference^15)^. Given the limited number of events, we avoided extensive multivariable adjustment; therefore, residual confounding by primary site cannot be excluded. Cisplatin eligibility itself may confound the association between muscle function and survival, because cisplatin ineligibility is commonly driven by impaired renal function, poorer performance status, and comorbidities. In our cohort, carboplatin-based therapy was more frequent in the impairment group, which may reflect underlying vulnerability rather than treatment effect alone.

BIA-derived estimates may be influenced by hydration status and measurement conditions, underscoring the need for standardized protocols^12)^. Although walking speed was assessed in all patients, measurement conditions in routine practice may vary, which may affect classification and supports the need for standardized assessment procedures across institutions^4)^. In addition, detailed information on chemotherapy regimens, dose intensity, and treatment-related toxicity was not incorporated into the present analysis and should be evaluated in future studies.

Future research should validate these findings in prospective multicenter cohorts, assess whether BIA plus grip strength improves prognostic stratification beyond established clinical factors, and determine whether longitudinal changes during treatment add predictive value^3, 4)^. Interventional studies that test supportive strategies guided by muscle function screening may also be warranted.

## Conclusions

Muscle function impairment identified by BIA and simple bedside tests was associated with poorer survival in chemotherapy-treated metastatic urothelial carcinoma. These findings are hypothesis-generating and support further evaluation of feasible frailty screening strategies in larger cohorts.

## Data availability

De-identified data supporting the findings of this study are available from the corresponding author upon reasonable request, subject to institutional and ethical restrictions.

## Author contributions

NM conceived the study, curated the data, performed the analyses, and drafted the manuscript. SH supervised the study and critically revised the manuscript. KK, SH, FK, KA, CT, KH, IS, and ID contributed to data interpretation and manuscript revision. All authors read and approved the final manuscript.

## Conflicts of interest statement

The authors declare that there are no conflicts of interest

## Supplementary Material

**Supplemental Figure 1:**
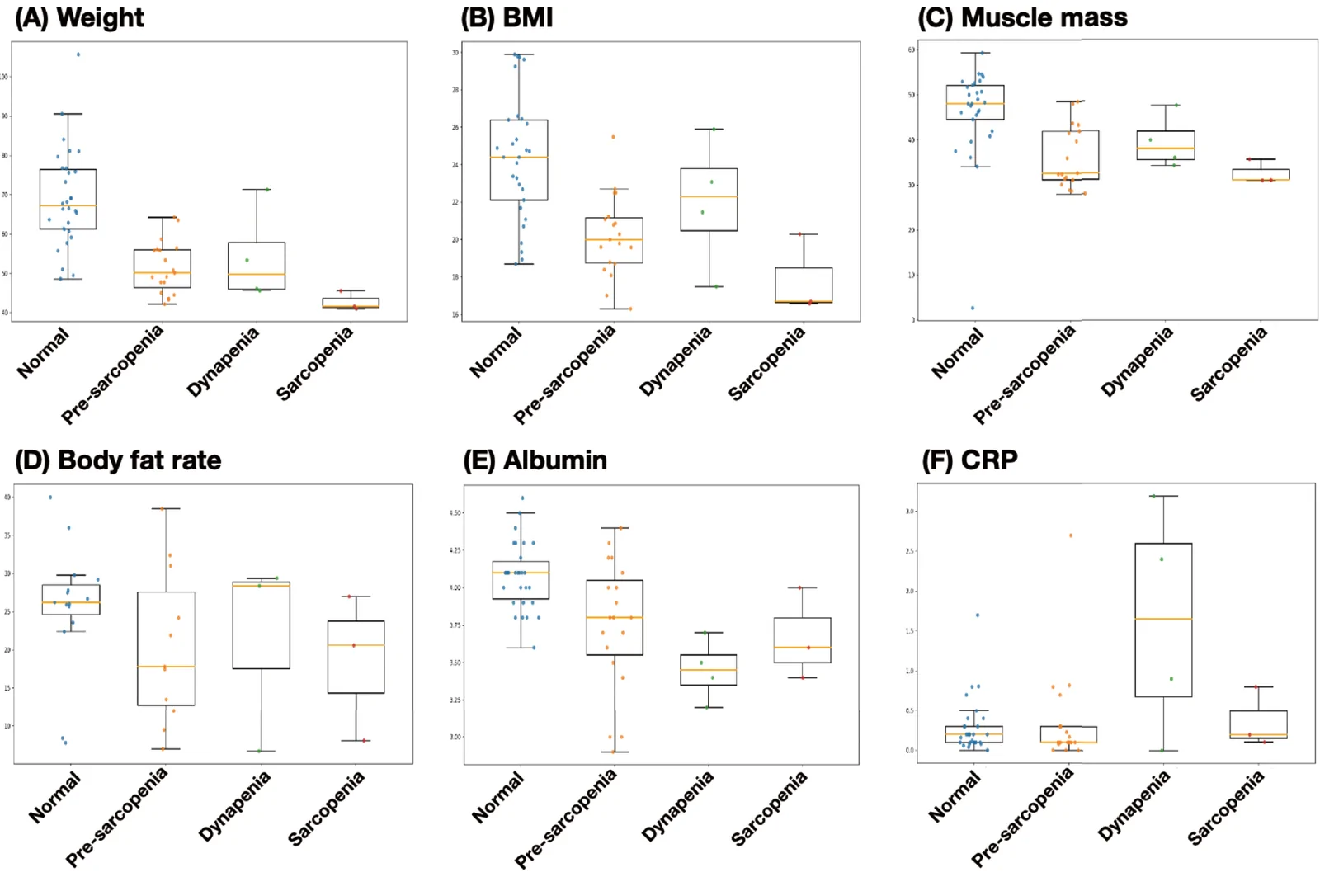
Body composition and laboratory measures by sarcopenia-related category Box-and-jitter plots show baseline values stratified by sarcopenia-related category (normal, pre-sarcopenia, dynapenia, and sarcopenia). Boxes indicate the median and interquartile range; whiskers represent the range, and points represent individual patients with jitter to avoid overlap. Panels show (A) body weight, (B) BMI, (C) lean body mass, (D) body fat rate, (E) serum albumin, and (F) C-reactive protein.

**Supplemental Figure 2:**
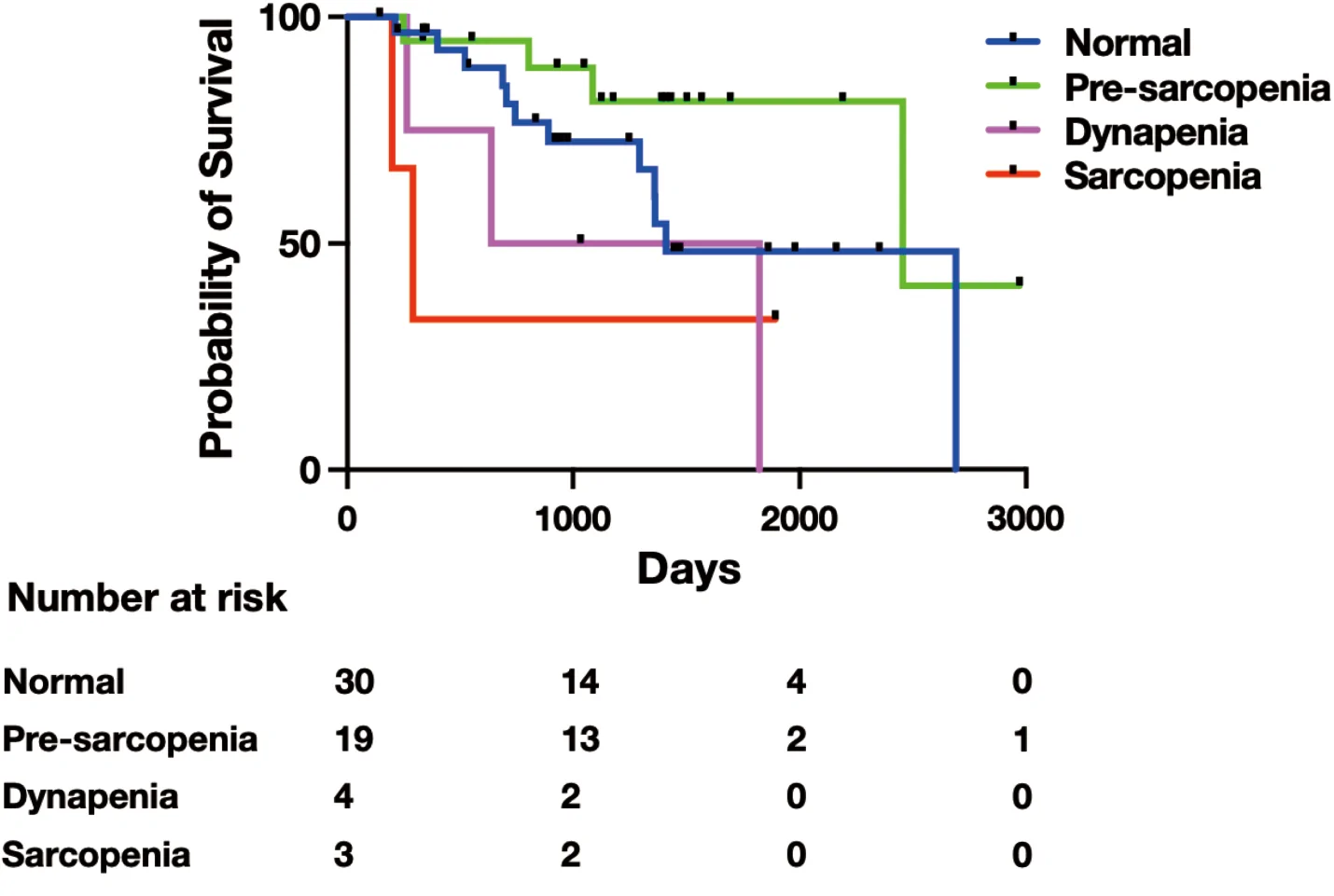
Overall survival by sarcopenia-related category Kaplan-Meier curves show overall survival across the four sarcopenia-related categories (normal, pre-sarcopenia, dynapenia, and sarcopenia). Curves are presented for descriptive purposes because the dynapenia and sarcopenia groups were small. Numbers at risk are shown below the x-axis.

**Supplemental Table 1:** 

Patient group for men	Grip strength (kg)	Walking speed (m/sec)	SMI (kg/m^2^)
Normal	≥ 28	> 1.0	≥ 7.0
Pre-sarcopenia	≥ 28	> 1.0	< 7.0
Dynapenia	< 28	< 1.0	≥ 7.0
Sarcopenia	< 28	< 1.0	< 7.0
			
Patient group for women	Grip strength (kg)	Walking speed (m/sec)	SMI (kg/m^2^)
Normal	≥ 18	> 1.0	≥ 5.7
Pre-sarcopenia	≥ 18	> 1.0	< 5.7
Dynapenia	< 18	< 1.0	≥ 5.7
Sarcopenia	< 18	< 1.0	< 5.7
